# Relationship between Associated Neuropsychological Factors and Fall Risk Factors in Community-Dwelling Elderly

**DOI:** 10.3390/healthcare10040728

**Published:** 2022-04-14

**Authors:** DongHyun Yi, SeungJun Jang, JongEun Yim

**Affiliations:** 1 Department of Physical Therapy, Graduate School of Sahmyook University, Seoul 01795, Korea; yidonghyun89@daum.net (D.Y.); tmdwns18@naver.com (S.J.); 2 Department of Physical Therapy, Sahmyook University, Seoul 01795, Korea

**Keywords:** elderly, fall, cognitive function, depression, fear of falls, fall risk factors

## Abstract

This study examined whether neuropsychological factors could affect fall risk factors in the community-dwelling elderly via correlation analysis. A total of 393 older adults (76.69 ± 6.01) participated in this study. Cognitive function, depression, fall efficacy, balance confidence, balance, gait, and muscle strength were evaluated, and the correlation between psychological factors and fall risk factors was analyzed. Additionally, a multiple regression analysis was conducted to determine whether or not there was a significant effect between psychological factors and fall risk factors. Analysis showed that the psychological factors examined were all significantly correlated with the fall risk factors. A correlation analysis between cognitive function and fall risk factors showed that the correlation coefficient of the 6-Meter Walk Test was highest; for depression and fall risk factors, the correlation coefficient of gait speed was highest; for fall efficacy and fall risk factors, the correlation coefficient of the 6-Meter Walk Test was highest; and for confidence in balancing and fall risk factors, the correlation coefficient of the 6-Meter Walk Test was highest. This study suggests that psychological factors affect fall risk factors in the community-dwelling elderly, and a multifaceted approach that includes psychological factors would be helpful in providing interventions for falls.

## 1. Introduction

Around 37.3 million falls occur worldwide each year, and most people aged 65 years and over experience falls [1]. Thirty percent of elderly people fall at least once each year, and this trend increases to 42% among people aged 70 years or older [2]. Additionally, a study reported that more than one-half of all unintentional injury-related deaths among adults aged 65 years and over are due to falls [3].

Such unintentional falls can lead to serious injuries and even death in the elderly [4]. Approximately 30–50% of falls cause minor injuries, such as bruises or contusion, but 5–10% of falls lead to major injuries, such as fractures and traumatic brain injury [5]. Additionally, even if a fall does not cause significant physical injury, it may lead to fear of falling, loss of self-confidence, and ambulation limitation, resulting in the inability to lead an independent life, social isolation, and deterioration of health [6]. Consequently, the occurrence of falls in elderly people is affected by both physical and psychological factors [7].

Falls are more frequent among elderly people because physiological changes associated with aging cause a ten-fold increase in the risk of falls compared to other age groups [8]. Most falls are not caused by a single risk factor, but rather by the interaction of various risk factors. Fall risk factors can be divided into environmental, extrinsic, and intrinsic factors. Among them, the intrinsic factors that affect falls most can be reclassified into physical and psychological factors [9]. Physical factors include weakened lower extremity muscle strength, decreased gait ability, decreased balance ability, decreased grip strength, decreased sensation, and decreased sensorimotor control [4,10]. To identify such physical risk factors, instruments with proven effectiveness and validity have been widely used in clinical practice [11,12]. In particular, balance ability is recognized as an important predictor of falls [13,14,15], and balance tests for elderly people include the 10-Meter Walk Test, Timed Up and Go Test (TUG), Functional Reach Test (FRT), Step Test, Berg Balance Scale (BBS), and One Leg Standing Test [16]. Psychological changes, such as fear of falling, depression, and cognitive decline, are considered other risk factors that increase the risk and incidence of falls [17]. Psychological factors include fear of falling, self-efficacy, mental health, emotional functioning, and life satisfaction [18,19,20,21,22].

Although studies regarding falls in the general elderly are being actively conducted, studies involving elderly people with cognitive impairment, mental illness, or disability remain scarce. This is because fall risk factors in such individuals are quite different. Most studies regarding fall prevention programs have been designed for physical improvement and physical weakness prevention in elderly people without mental impairment. Consequently, current studies on fall prevention have failed to prevent falls in elderly people with mental disabilities [23], indicating that various factors might affect this specific group [24]. As the number of risk factors increases, the frequency of falls increases, and the incidence of resulting injuries also increases [25,26,27]. Therefore, a multifaceted approach to fall prevention is required [28]. Falls are highly associated with cognitive levels, and lower cognitive levels are associated with a higher probability of falling [29,30]. Additionally, falls may cause a fear of falling and post-fall anxiety as well as physical trauma, increasing depression and social isolation, and lowering fall self-efficacy [31]. Therefore, it is critical to develop fall prevention and prediction programs that consider both physical and psychological factors, and it is also necessary to identify fall risk factors among various psychological factors. Most current studies regarding falls have focused on improving physical function, but studies on the psychological factors for falls, which should also be considered important, are lacking.

This study aimed to analyze the relationship between mental factors and fall risk factors among community-dwelling elderly people, and to investigate whether both neuropsychological factors and physical factors among various fall risk factors can affect the risk of falls. This study also aimed to present the need for a multifaceted approach involving neuropsychological factors for the development of fall prevention programs and basic data.

## 2. Materials and Methods

This correlation study aimed to measure neuropsychological factors such as cognitive function, depression, fear of falling, and fall risk factors, such as gait, balance, and muscle strength in 404 community-dwelling elderly people aged 65 or older, and to investigate the relationship between neuropsychological factors and fall risk in elderly people.

### 2.1. Participants

Among the 404 individuals assessed, the final participants selected for this study were 393 elderly persons aged 65 years or older, who visited a senior welfare center located in Gwangjin-gu, Seoul, a senior welfare center located in Mokpo city, Jeollanam-do, a senior citizen center located in Kongju city, and a senior welfare center located in Pocheon city, Gyeonggi-do, South Korea, and who voluntarily agreed to participate in this study. The mean age of the participants was 76.5 years, and those who were able to walk without the use of ambulatory assistive aids (walkers, canes, etc.) were able to communicate, were able to live independently, and were included in this study. Among them were those who were taking drugs that could affect balance and drugs related to psychological disorders, those with neurological disorders such as anxiety and depression, those with musculoskeletal disorders, those with physical activity limitations due to personal injury or disease, and those with severe visual or sensory impairment; those with scores under 20 in MMSE were excluded from this study. Before evaluation, all participants were fully informed of the purpose and methods of this study, and only those who provided consent were evaluated. 

### 2.2. Instruments

#### 2.2.1. Korean Mini-Mental State Examination (K-MMSE)

Cognitive function was measured using the Korean version of the Mini-Mental State Examination (K-MMSE), which was originally developed by Folstein et al. [32] and standardized for Korean elderly people by Kwon and Park [33]. The K-MMSE has a total score of 30 points, including 5 points for time orientation, 5 points for place orientation, 3 points for memory registration, 3 points for recall, 5 points for attention and calculation, 7 points for language skills, and 2 points for understanding and judgment. A higher score indicates higher cognitive function; a score of ≤20 points indicates definitive cognitive impairment, a score of 21–23 points indicates suspected cognitive impairment, and a score of ≥24 points indicates definitive normal. Additionally, if a participant had no formal education or was illiterate, a score was determined by adding 1 point to the score for time orientation, 2 points to the score for attention and calculation, and 1 point to the score for language skills; however, it was ensured that the score for each item did not exceed the maximum score, in accordance with scoring rules [33]. Those with a score of ≤20 points were excluded from this study. 

#### 2.2.2. Korean Geriatric Depression Scale (GDS)

Depression was measured using the Korean Geriatric Depression Scale, which was developed by Yesavage et al. [34] and standardized by Jung et al. [35]. The GDS has a yes/no response format in which participants are asked to respond by answering yes or no through a self-report questionnaire or verbal questions, and a yes response to each item is scored 1 point. A score of 14–18 points (out of 30 points) indicates mild depression, a score of 19–21 points indicates moderate depression, and a score of ≥22 points indicates severe depression. It consists of a total of 30 items, with a higher score indicating more severe depression.

#### 2.2.3. Korean Version of Falls Efficacy Scale

Fall efficacy was measured using the Korean version of the Falls Efficacy Scale (FES), which was developed by Tinetti et al. [36] and standardized by Jang et al. [37]. This tool is designed to directly check an individual’s confidence in his/her ability to perform each of 10 daily activities without falling. The score for each item ranges from 1 to 10 points. A higher score indicates greater confidence, and a lower score indicates lower confidence. Fall efficacy in the participants is assessed based on the sum of the scores for a total of 10 items. The total score ranges from a minimum of 10 points to a maximum of 100 points, and a higher total score indicates a higher efficacy for falls [31].

#### 2.2.4. Activities-Specific Balance Confidence Scale (ABC)

Balance confidence was measured using the Korean version of the Activities-specific Balance Confidence Scale (ABC), which was originally developed by Power et al. [38] and standardized by Jang et al. [37]. The ABC measures balance confidence according to location and various activities and consists of a total of 16 activities. This tool is designed to determine balance confidence by asking elderly people about their confidence in how well they can perform activities without falling or losing their balance. The score for each item ranges from 0% to 100%. A higher score indicates greater confidence. The average score for the 16 items is a total score.

#### 2.2.5. Static Balance Ability

1. One Leg Stand Test: Static balance ability was measured using the One Leg Stand Test, which assesses an individual’s ability to maintain an upright balance when the base of the support is reduced [39]. The One Leg Stand test is intended to measure an individual’s ability to maintain an upright posture against gravity on a fixed support surface. For measurement, each participant, keeping their eyes open and their hands on their waists, was asked to stand on one leg at the researcher’s verbal “Go” and maintain the posture. The length of time that each participant maintained the posture on one leg was measured twice, and the maximum value was used.
2. Sharpened Romberg’s Test: Static balance ability was assessed using the Sharpened Romberg’s Test. In this test, each participant was asked to position their dominant foot behind their non-dominant foot and stand in a heel-toe position with arms crossed over their chest. The test was performed with both, eyes open and eyes closed, and the stopwatch (timing) was stopped when each participant opened their eyes and moved or maintained the position for more than 60 s. Three trials were performed, and the best record was selected for use. If the time the position was maintained in the first and second trials exceeded 60 s, a third trial was not performed [40].

#### 2.2.6. Dynamic Balance Ability

1. Timed Up and Go Test (TUG): Dynamic balance ability was measured using the TUG test [41]. The TUG begins with the participant seated leaning back in a chair with armrests. When the test starts, the participant was asked to stand up from the chair, walk a distance of 3 m, turn around the turning point, walk back, and return to the seated position. The time taken from the start to the seated position was measured. Physical support or help from therapists was limited. Measurements were performed three times, and the mean score for the values obtained from the three measurements was used. During the measurement, participants were allowed to use their usual shoes or assistive aids.
2. Four Square Step Test (FSST): Dynamic balance ability was measured using the FSST. The FSST is a tool involving stepping over four canes, each 2.5 cm high and 90 cm long, placed at a 90° angle on the floor in a + (‘plus’) configuration. Each participant stands in Square 1, one of the four squares facing forward. The participant steps over each cane in a clockwise direction to take one turn and then goes back in the opposite or counterclockwise direction. The trial starts when the first foot makes contact with the floor in Square 2. If the participant made contact with a cane or lost balance, or both feet did not make contact with the floor in each square, the trial was repeated. After three measurements were made, the mean value was used as the result value [42].

#### 2.2.7. Gait

1. 6-Meter Walk Test: Gait ability was measured using the 6-Meter Walk Test (6 MWT). The time taken for the participant to start in a standing position and walk a distance of 6 m at the researcher’s verbal “Go” was measured. Measurements were repeated three times and the mean values were used. During the 6 MWT, the participants were allowed to use assistive aids and were recorded so that they could continuously use assistive aids for further assessments [43].
2. Gait speed: Gait speed was measured using a gait analyzer (OptoGait, Microgate S.r.l, Bolzano, Italy). The gait analyzer consists of two receiving bars with a length of 1 m and a webcam (Logitech Webcam Por 9000), and 96 light-emitting diodes (LEDs) are installed at an interval of 1 m on the two bars, which transmit and receive data through infrared light. The optical sensor transmits and receives data at a frequency of 1000 Hz. While the participants walk between two parallel bars, the gait analyzer measures double support, gait cycle, stride length, gait speed, and cadence.

#### 2.2.8. Muscle Strength

1. Grip strength: The muscle strength of the upper extremities was measured using a dynamometer (Jamar hydraulic dynamometer, Model 5030J1, Salt Lake, UT, USA). After each participant bent the elbow joint of the respective arm at a 90° angle in a sitting position in a chair, he/she held a dynamometer to measure grip strength. Grip strength was measured twice for the left and right hands, and the highest measure was used [44].
2. Five Times Sit-To-Stand (5TSTS): The muscle strength of the lower extremities was measured using the Five Times Sit-to-Stand (5TSTS) test. Participants were asked to sit on a chair with no armrests and with a backrest, and keep their arms folded across their chest. The timing started once each participant’s back left the backrest of the chair. Each participant stood up completely (knee and waist were completely extended), sat down (touched the chair backrest) five times, and the total time taken from the start to returning to sitting after the fifth rise was measured [45].

### 2.3. Data Analysis

All data obtained from this experiment were analyzed using the SPSS/PC 12.0 program. Pearson’s correlation analysis was used to analyze the relationships between neuropsychological factors and fall risk factors. Stepwise multiple regression analysis was performed to determine whether there was a significant relationship between neuropsychological factors and fall risk factors. Fall risk factors were used as independent variables, and the K-MMSE score, GDS score, the Korean Fall Efficacy Scale (FES) score, and the ABC score were used as the dependent variables. All statistical significance levels were set at *p* ≤ 0.05.

## 3. Results

### 3.1. General Characteristics and Fall-Related Characteristics of the Participants

The general characteristics of the participants are shown in Table 1. Of the participants, 76.6% (301 persons) were women and 23.4% (*n* = 92) were men, and the mean age was 76.69 ± 6.01 years. Of the participants, 74.8% (*n* = 294) had experienced at least one fall, and the average number of falls in those who experienced at least one fall in the last year was 2 ± 1.17.

### 3.2. Relationship between Neuropsychological Factors and Fall Risk Factors

The results of the analysis of the relationships between the variables are shown in Table 2. Analysis was performed to determine the relationship between neuropsychological factors, such as cognitive function (K-MMSE), depression (KGDS), fall efficacy (FES), balance confidence (ABC), and fall risk factors such as gait (6-Meter Walk Test), balance (Sharpened Romberg’s Test with eyes open; Sharpened Romberg’s Test with eyes closed; TUG; One Leg Stand (right); One Leg Stand (left); FSST), muscle strength (5TSTS; grip strength (left); grip strength (right); and gait speed).

### 3.3. Effects of Fall Risk Factors on Neuropsychological Factors

#### 3.3.1. Association between Fall Risk Factors and Cognitive Factors 

The results of the analysis revealed that R^2^ = 0.266, indicating an explanatory power of 26.6%. Among the fall risk factors as independent variables, Sharpened Romberg’s Test (eyes open), Sharpened Romberg’s Test (eyes closed), TUG, grip strength (left), One Leg Stand (left), One Leg Stand (right), and gait speed were not significantly related to cognitive function. Moreover, 6-Meter Walk Test, grip strength (right), FSST, and 5TSTS had significant effects on cognitive function. Among them, FSST had the greatest effect (β = −0.397) on cognitive function, followed by 5TSTS (β = 0.299), 6-Meter Walk Test (β = −0.256), and grip strength (right) (β = 0.208) (Table 3).

#### 3.3.2. Effects of Fall Risk Factors on Depression

The analysis results revealed that R^2^ = 0.155, indicating an explanatory power of 15.5%. Among fall risk factors as independent variables, the 6-Meter Walk Test, FSST, TUG, Sharpened Romberg’s Test (eyes open), grip strength (left), One Leg Stand (left), One Leg Stand (right), and 5TSTS were not significantly associated with depression. However, gait speed, Sharpened Romberg’s Test (eyes closed), and grip strength (right) had significant effects on depression. Among them, gait speed had the greatest effect (β = −0.254) on depression, followed by Sharpened Romberg’s Test (eyes closed) (β = −0.125), and grip strength (right) (β = −0.136) (Table 4).

#### 3.3.3. Association between Fall Risk Factors and Fall Efficacy 

The analysis results revealed that R^2^ = 0.227, indicating an explanatory power of 22.7%. Among the fall risk factors as independent variables, FSST, TUG, Sharpened Romberg’s Test (eyes open), Sharpened Romberg’s Test (eyes closed), grip strength (left), One Leg Stand (left), gait speed, and 5TSTS had no significant relationship with fall efficacy. However, the 6-Meter Walk Test, grip strength (right), and One Leg Stand (right) had significant effects on fall efficacy. Among them, the 6-Meter Walk Test had the greatest effect (β = 0.335) on fall efficacy, followed by grip strength (right) (β = −0.174), and One Leg Stand (right) (β = −0.100) (Table 5). 

#### 3.3.4. Association between Fall Risk Factors and Balance Confidence 

The analysis results revealed that R^2^ = 0.320, indicating an explanatory power of 32.0%. Among the fall risk factors as independent variables, FSST, TUG, Sharpened Romberg’s Test (eyes open), Sharpened Romberg’s Test (eyes closed), grip strength (left), One Leg Stand (left), and 5TSTS had no significant effect on balance confidence. However, the 6-Meter Walk Test, One Leg Stand (right), grip strength (right), and gait speed had significant effects on fall efficacy. Among them, the 6-Meter Walk Test had the greatest effects (β = −0.306) on balance confidence, followed by One Leg Stand (right) (β = 0.133), grip strength (right) (β = 0.099), and gait speed (β = 0.166) (Table 6). 

## 4. Discussion

Cognitive function among elderly people is closely related to motor ability, and cognitive function among elderly people decreases with age [46]. Those with poorer scores for place orientation, attention, and calculation, and visuospatial function among cognitive functions in the MMSE were 1.7 to 2 times more likely to fall [30]. Therefore, cognitive function may also be associated with fall risk factors, and an improvement in cognitive function may lead to reduced falls. The results of analyzing the relationship between cognitive function and fall risk factors in this study revealed that the relationship coefficients of the 6-Meter Walk Test, gait speed, and FSST were high. Among the fall risk factors affecting cognitive function as determined by multiple regression analysis, FSST had the greatest effect (β = −0.397) on cognitive function. FSST is cognitively more demanding, requiring participants to remember and complete a series of steps. FSST is used for PD patients because completing the FSST may reveal deficits related to divides attention between mental processing and postural control [47]. These results are consistent with the results of a study by Salbach et al. [48], who reported that impaired balance is strongly related to cognition and that lower balance is associated with lower cognitive function. The results of this study showed that there was a relationship between cognitive function and fall risk factors, which supported the results of a study by Ramirez et al. [30] showing that falls can be predicted through MMSE scores; the results of a study by Muir et al. [29] showed that lower cognitive levels were associated with a higher likelihood of falls. As such, cognitive function is highly associated with fall risk factors, and it is assumed that the development and implementation of interventions for enhancing cognitive function among elderly people will help to reduce the number of falls in the elderly.

Changes in depression are independently related to fall rates [49] and may affect falls directly or through chronic pain, disease, antipsychotic drugs, functional limitations, and cognitive decline [49,50]. However, in general, depression is difficult to detect and is not well treated in primary care practice [51]. The early detection and treatment of depression in elderly people at risk of falling may affect the prevention of falls, and the prevention and treatment of falls and depression are critical to improving elderly people’s health and welfare [52]. The results of analyzing the relationship between depression and fall risk factors in this study revealed that the relationship coefficients of gait speed, 6-Meter Walk Test, and FSST among elderly people were high. With regard to the fall risk factors affecting depression as determined by multiple regression analysis, gait speed was found to have the greatest effect on depression (β = −0.254). Compared to older adults, depressed subjects showed decreased stride length and double limb support related to slower gait speed [53]. A study by Serrano-Checa et al. [54] involving elderly women indicated that depression had an inversely proportional relationship with dynamic balance, decreased sleep ability, and severe depression, thus decreasing functional mobility, leading to an increased risk of falls. The results of this study found that GDS was highly correlated with gait speed, 6 MWT, which measured gait ability, and FSST, which measured dynamic balance. These results are similar to those of the study by Serrano-Checa et al. [54], in which depression was measured using a different scale. A study by Lin et al. [55] regarding geriatric depression and the quality of life in elderly people described that reducing depressive mood in the elderly could improve the quality of life and reduce the fall rate. Therefore, to accurately evaluate falls in elderly people, it is necessary to evaluate not only functional factors but also anxiety and depression.

Fear of falling is defined as ongoing worry about falling that limits the performance of activities and causes additional falls [56]. A fall is an independent predictor of the fear of falling, and the fear of falling is a predictor of falls [57]. Such fear of falling may also be present in people who have never experienced a fall [58]. Therefore, the fear of falling is closely related to falls, and preventing falls before they occur will lead to fewer falls among elderly people. As reported in a recent study analyzing the causal relationship between fall self-efficacy (ABC) and physical activity levels [59], and another study regarding the relationship between fall efficacy, functional performance, and psychological factors [60], active discussions about functional and psychological factors for falls are underway. The results of analyzing the relationship between fall efficacy and fall risk factors in this study revealed that the correlation coefficients of the 6-Meter Walk Test, gait speed and TUG were high. With regard to the fall risk factors affecting fall efficacy as determined by multiple regression analysis, the 6-Meter Walk Test had the greatest effect (β = 0.335) on fall efficacy. 

Additionally, the results of analyzing the relationship between balance confidence and fall risk factors revealed that the relationship coefficients of the 6-Meter Walk Test, gait speed and TUG were high. With regard to the fall risk factors affecting balance confidence as determined by multiple regression analysis, the 6-Meter Walk Test had the greatest effect (β = −0.306) on balance confidence. Anxious older adults walk more slowly, have shorter strides, longer double-limb support times, and higher gait variability [61]. These findings are consistent with the results of a study by Cromwell and Newton [62], which indicated that there was a strong relationship between balance confidence and gait speed. Self-efficacy is considered to be a very important factor for gait ability in elderly people and greatly affects various physical motor abilities, including social participation [63]. Furthermore, the correlation coefficients of fall efficacy and balance confidence with TUG, a dynamic balance measure, were high at 0.362 and −0.394, respectively, which were consistent with the results of studies by Figueiredo and Neves [64], and Mirelman et al. [65], who reported a significant relationship between TUG score and anxiety. This study found a strong relationship between neuropsychological factors and fall risk factors, which were balance measures such as One Leg Stand and TUG. These results are consistent with the results of a study by Maki et al. [66] using the same scale. Cleary and Skornyakov [42] investigated whether elderly people had a fall in 6 months by dividing elderly people into two groups according to balance confidence, and stated that balance confidence could predict future falls in community-dwelling elderly people. As such, the fear of falling has a relationship with balance, muscle strength, and gait speed, which should be considered as predictors of falls.

Furthermore, a study by Adamek and Yoder-Slater [67] reported that, when the fear of falling in elderly people increased, GDS scores and depression also increased. Furthermore, a study by Rivasi et al. [68] reported that the fear of falling could be predicted based on depression, and that the fear of falling was highly associated with depression. Considering these results, it can be inferred that depression may significantly affect the relationship between the fall efficacy scale and balance confidence, and further studies regarding the association between fall efficacy and balance confidence are needed. 

Gait speed has been repeatedly studied as a reliable tool for predicting falls and discriminating between falls and non-falls [69]. Among the fall risk factors used in this study, the 6-Meter Walk Test and gait speed showed a high relationship with the MMSE, depression, fall efficacy, and balance confidence. These findings show that gait ability has a stronger relationship with neuropsychological factors compared to other fall risk factors, and that gait ability in elderly people is most closely related to neuropsychological factors. These results support the results of a previous study [70], which showed that decreased walking speed, decreased mobility, and impaired dynamic balance were associated with an increased risk of falls in community-dwelling elderly people [70]. Because the participants in this study had no functional defects affecting gait speed, most of them showed a gait speed of ≥1.0 m/s. This indicates that neuropsychological factors should be considered important in fall prevention gait training for elderly people who can perform activities of daily living on their own.

As community-based physical therapy practice expands, falls are one of the intervention goals that should be considered essential for community-dwelling elderly people. However, in the current clinical physical therapy practice, most fall-related intervention modalities have been limited to physical factors, and fall prevention programs and education are also focused on physical factors rather than neuropsychological factors. In particular, the important role of physical therapists is to have a comprehensive understanding of falls as well as physical and cognitive factors related to falls in elderly people, removing fall risk factors that may be present, and providing them with related education and training. This study found that fall risk factors were closely related to neuropsychological factors such as cognitive state, depression, and fear of falling. Therefore, when providing interventions for mitigating fall risk factors, it is thought that a multi-faceted approach that considers neuropsychological factors will be helpful in providing better interventions for elderly people. Although the number of subjects is large, since it is a cross-sectional study, it has limitations in causal inference. As a cross-sectional study, it was insufficient to prove the cause–effect relationship of each factor. In future studies, it is necessary to prove the relationship between falls and neuropsychological factors through a prospective cohort study.

## 5. Conclusions

This study aimed to investigate the relationship between neuropsychological factors and fall risk factors in community-dwelling elderly people, and to determine whether the neuropsychological factors among various factors for falls can affect the risk of falls. The results of the study revealed that four neuropsychological factors—cognitive function, depression, fall efficacy, and balance confidence—were all significantly correlated with fall risk factors. It was also found that balance confidence was more closely correlated with fall risk factors than with other neuropsychological factors. 

Additionally, for cognitive function, depression, and fear of falling presented in this study, other psychological factors affecting fall risk factors should be considered in future studies. Such psychological factors include psychological changes associated with drugs, sleep patterns, and social isolation. Further studies are needed to determine the relationships between neuropsychological factors, physical factors, and environmental factors. Multifaceted studies regarding falls are also needed to effectively prevent falls among elderly people. Additionally, the development of evaluation tools that can assess all these factors is required. 

## Figures and Tables

**Table 1:** General characteristics of the subjects (*n* = 393).

Variables		Subject
Gender	Male	92 (23.4%)
	Female	301 (76.6%)
Age	(years)	76.69 ± 6.01
Fall experience	Yes	294 (74.8%)
	No	99 (25.2%)
Fall frequency	(Frequency)	2 ± 1.17

**Table 2:** A comparison table of the correlation between mental factors and fall risk factors (*n* = 393).

Variables	6MWT	SRT EO	SRT EC	TUG	5TSTS	FSST	Grip Strength Left	Grip Strength Right	OLS R	OLS L	Gait Speed
MMSE	−0.418 **	0.203 **	0.169 **	−0.246 **	−0.186 **	−0.395 **	0.318 **	0.348 **	0.251 **	0.248 **	0.399 **
GDS	0.340 **	−0.257 **	−0.235 **	0.252 **	0.229 **	0.266 **	−0.220 **	−0.263 **	−0.184 **	−0.158 **	−0.342 **
FES	0.441 **	−0.249 **	−0.176 **	0.362 **	0.256 **	0.285 **	−0.283 **	−0.336 **	−0.265 **	−0.245 **	−0.424 **
ABC	−0.534 **	0.283 **	0.178 **	−0.394 **	−0.321 **	−0.353 **	0.276 **	0.327 **	0.336 **	0.307 **	0.521 **

**Note.** 6MWT = 6 Meter Walk Test; SRT EO = Sharpened Romberg Test Eye Open; SRT EC = Sharpened Romberg Test Eye Close; TUG = Timed Up and Go test; 5TSTS = 5 Times Sit To Stand Test; FSST = Four Square Step Test; OLS R = One Leg Stand Right side; OLS L = One Leg Stand Left side; MMSE = Mini-Mental Status Examination; GDS = Geriatric Depression Scale; FES = Fall Efficacy Scale; ABC = Activities-specific Balance Confidence Scale. ** *p* < 0.01

**Table 3:** Association between Fall Risk Factors and Cognitive Factors (*n* = 393).

Variables	Non-Standard Coefficient		Standard Coefficient	*t*(*P*)	*VIF*
	*B*	*SE*	*β*		
6MWT	−0.475	0.119	−0.256	−3.993 (0.000)	2.195
Right Grip strength	0.093	0.021	0.208	4.396 (0.000)	1.196
FSST	−0.356	0.066	−0.379	−5.358 (0.000)	2.670
5TSTS	0.180	0.039	0.299	4.577 (0.000)	2.267

**Note.** 6MWT = 6 Meter Walk Test; FSST = Four Square Step Test; 5TSTS = 5 Times Sit To Stand Test. **Dependent variable:** MMSE (Mini-Mental State Examination). **Excluded variables**: Sharpened Romberg Test Eye Open, Sharpened Romberg Test Eye Close, Timed Up and Go test, Left Grip strength, One Leg Stand Right side, One Leg Stand Left side, gait speed.

**Table 4:** Association between fall risk factors and depression (*n* = 393).

Variables	Non-Standard Coefficient		Standard Coefficient	*t*(*P*)	*VIF*
	*B*	*SE*	*β*		
Gait speed	−7.099	1.440	−0.254	−4.931 (0.000)	1.230
SRT EC	−0.051	0.015	−0.166	−3.500 (0.001)	1.045
Right Grip strength	−0.125	0.047	−0.136	−2.675 (0.008)	1.203

**Note.** SRT EC = Sharpened Romberg Test Eye Close. **Dependent variable:** GDS (Geriatric Depression Scale). **Excluded variables:** 6 Meter Walk Test, Four Square Step Test, Timed Up and Go test, Sharpened Romberg Test Eye Open, Left grip strength, One Leg Stand Left side, One Leg Stand Right side, 5 Times Sit To Stand Test.

**Table 5:** Association between fall risk factors and fall efficacy (*n* = 393).

Variables	Non-Standard Coefficient		Standard Coefficient	*t(P)*	*VIF*
	*B*	*SE*	*β*		
6MWT	1.436	0.217	0.335	6.615 (0.000)	1.294
Right Grip strength	−0.179	0.051	−0.174	−3.533 (0.000)	1.223
OLS R	−0.053	0.025	−0.100	−2.075 (0.039)	1.169

**Note.** 6MWT = 6 Meter Walk Test; OLS R = One Leg Stand Right side. **Dependent variable:** FES (Falls Efficacy Scale). **Excluded variables:** Four Square Step Test, Timed Up and Go test, Sharpened Romberg Test Eye Open, SharpenedRomberg Test Eye Closed, Left grip strength, One Leg Stand Left side, gait speed, 5 Times Sit To Stand Test.

**Table 6:** Association between fall risk factors and balance confidence (*n* = 393).

Variables	Non-Standard Coefficient		Standard Coefficient	*t(* *P* *)*	*VIF*
	*B*	*SE*	*β*		
6MWT	−55.383	14.411	−0.306	−3.843 (0.000)	3.657
OLS R	2.963	1.020	0.133	2.904 (0.004)	1.216
Right Grip strength	4.289	2.001	0.099	2.144 (0.033)	1.236
Gait speed	219.530	107.398	0.166	2.044 (0.042)	3.818

**Note.** 6MWT = 6 Meter Walk Test; OLS R = One Leg Stand Right side. **Dependent variable:** ABC (Activities-specific Balance Confidence Scale). **Excluded variables:** Four Square Step Test, Timed Up and Go test, Sharpened Romberg Test Eye Open, Sharpened Romberg Test Eye Closed, Left grip strength, One Leg Stand Left side, 5 Times Sit To Stand Test.

## Data Availability

Not applicable.
